# Characterizing laser-plasma ion accelerators driving an intense neutron beam via nuclear signatures

**DOI:** 10.1038/s41598-019-39054-z

**Published:** 2019-02-14

**Authors:** A. Favalli, N. Guler, D. Henzlova, S. Croft, K. Falk, D. C. Gautier, K. D. Ianakiev, M. Iliev, S. Palaniyappan, M. Roth, J. C. Fernandez, M. T. Swinhoe

**Affiliations:** 1 Los Alamos National Laboratory, Los Alamos, New Mexico 87545 USA; 2 Spectral Sciences, Burlington, Massachusetts, 01803 USA; 3 ELI Beamlines, Institute of Physics of the ASCR, Na Slovance 2, Prague, 18221 Czech Republic; 4 Oak Ridge National Laboratory, 1 Bethel Valley Road, Oak Ridge, TN 37831 USA; 5 Institut für Kernphysik, Technische Universität Darmstadt, 64289 Darmstadt, Germany; 6 Helmholtz-Zentrum Dresden-Rossendorf, 01328 Dresden, Germany

## Abstract

Compact, bright neutron sources are opening up several emerging applications including detection of nuclear materials for national security applications. At Los Alamos National Laboratory, we have used a short-pulse laser to accelerate deuterons in the relativistic transparency regime. These deuterons impinge on a beryllium converter to generate neutrons. During the initial experiments where these neutrons were used for active interrogation of uranium and plutonium, we observed *β*-delayed neutron production from decay of ^9^Li, formed by the high-energy deuteron bombardment of the beryllium converter. Analysis of the delayed neutrons provides novel evidence of the divergence of the highest energy portion of the deuterons (i.e., above 10 MeV/nucleon) from the laser axis, a documented feature of the breakout afterburner laser-plasma ion acceleration mechanism. These delayed neutrons form the basis of non-intrusive diagnostics for determining the features of deuteron acceleration as well as monitoring neutron production for the next generation of laser-driven neutron sources.

## Introduction

Intense laser-driven ion beams have been the subject of considerable study for over a decade^1,2^. By exploiting advanced mechanisms of laser-driven ion acceleration, a new intense and short-duration neutron source with record flux (>10^10^ n/sr)^3,4^ has been demonstrated at the Trident laser facility at Los Alamos National Laboratory (LANL)^5^. In these experiments, neutrons are generated from a multistep process starting with the interaction of a short-pulse laser with a deuterated-plastic nanofoil target to make an intense beam of protons and deuterons. The ion beam subsequently impinges on a suitable converter material to drive the neutron beam. This source has the particularly useful properties of high intensity, short-duration and a forward peaked distribution. Laser-driven neutron sources offer an alternative path for the development of compact, bright and penetrating neutron sources for many applications^6–8^.

Laser-ion acceleration presently relies on very intense (*I* > 10^20^ W/cm^2^) laser fields on plasma targets to create collective effects that drive large accelerating electric fields with field strengths of tens of TV/m over very short distances (microns). The Trident laser facility provides a very high-contrast^9^ laser pulse of energy ~80 J, wavelength *λ*_0_ = 1053 nm, 600 fs FWHM duration, and peak on-target laser intensities up to 10^21^ W/cm^2^. The very high-contrast enables fielding plastic-foil targets (~1 g.cm^−3^) with thicknesses typically in the range of 300–700 nm. The target heats up rapidly during the main pulse rise and becomes relativistically transparent^10^ to the laser by the time of peak power. After transparency, the laser interacts volumetrically with the plasma and accelerates ions in the interaction region to high energies^11–17^.

Our experiments^4^ have demonstrated that the high-energy (up to about 80–100 MeV) deuterons produced in the relativistically transparent regime can create forward directed neutron emission primarily from deuteron break-up mechanisms in the converter, in addition to the prompt isotropic component from reactions such as ^9^Be(d, n)^10^B. One of the motivations for such a neutron source is the capability to perform an assay of special nuclear materials for nuclear materials accountancy, safeguards and national security applications. A penetrating intense neutron burst offers the potential of achieving a high signal-to-noise ratio in difficult environments (e.g., with high neutron background emitted by the interrogated item) and promises a short assay time, which translates to a high interrogated item throughput. This application, also known as active interrogation, is based on the measurement of induced neutron signatures to identify/assay nuclear materials during (detecting prompt fission neutrons) and after (detecting delayed neutrons from fission products) an interrogation with an external neutron pulse^18,19^.

During these groundbreaking experiments at the Trident^5^ laser facility, delayed neutron production from ^9^Li decay $({}^{9}Li\,\mathop{\longrightarrow }\limits^{{\beta }^{-}}{}^{8}Be+n)$^20^ was observed. The ^9^Li was unambiguously identified in our experiments by its characteristic half-life of 178.3 ms. The production of ^9^Li occurs in the Be converter and is attributed to two distinct nuclear reactions: ^9^Be(d, 2p)^9^Li^21^ and ^9^Be(n, p)^9^Li^22^. These reactions have energy thresholds of 18.42 and 14.26 MeV respectively, and the (d, 2p) reaction is the dominant channel. ^9^Li subsequently *β*-decays, with one branch producing delayed neutrons with the life time 1/e-value of *τ* = 257.2 ms^20^. Because of the energy thresholds, only the higher-energy portion of the deuteron spectrum above the threshold contributes to the production of the delayed neutrons.

In this paper, the results from delayed neutron production from ^9^Li decay and of the prompt neutron time-of-flight spectra along the beam axis observed in these experiments are presented and their implications are considered. Consequently important results are presented here for the first time. (1) We show that neutron production is a powerful, independent and complementary probe of the laser-driven deuteron beam. It enables experiments (as done here) to distinguish ion acceleration mechanisms and provides critical information for the design of a practical interrogation system. (2) The high-energy portion (>18 MeV) of the deuterium beam responsible for the novel intense neutron-beam source in refs^3,4^, made by the interaction of the laser beam with a flat nanofoil, is not emitted along the laser-axis but emerges at some angle in a ring or part of a ring (e.g.lobe). That rules out ion acceleration mechanisms widely studied by the community^1^, such as radiation-pressure acceleration (RPA) and target-normal sheath acceleration (TNSA), where the highest ion energies are observed on axis. It is qualitatively consistent with the Breakout Afterburner^13,23^, thus providing strong experimental evidence of one of its theoretically-identified signatures. However, we show that in this case, this feature is much more pronounced than previously thought based on limited measurements with ion spectrometers. (3) ^9^Li *β*-delayed neutrons have been identified as a source of delayed-neutron background that must be taken into account in the design of the new generation of laser-driven neutron sources for application such as assay of nuclear materials^6,7^.

## Results

**Figure 1:**
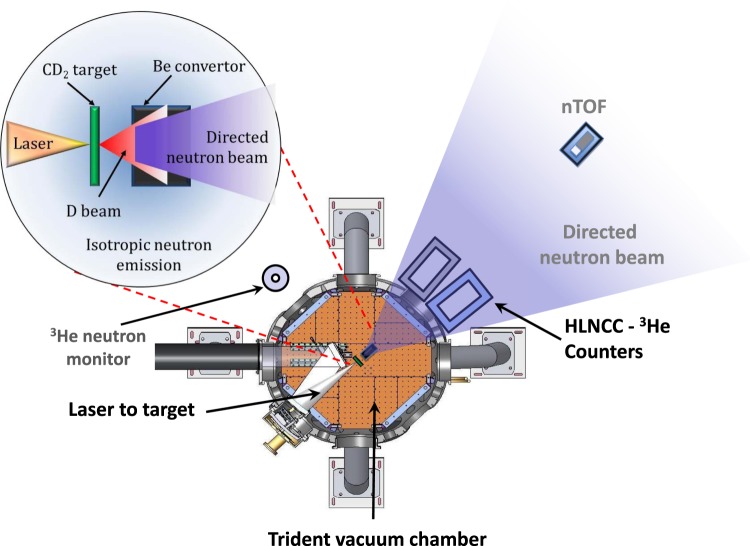
Experimental setup at Trident facility (not in scale).

Our experimental setup is shown in Fig. 1. The target chamber houses the laser focusing optics (in this experiment an F/3 parabolic mirror), the target (for this run, primarily ~350 nm thick deuterated polyethylene (CD_2_) foils) and the converter (a Be cylinder 20 mm diameter and 40 mm depth). All the diagnostic equipment was positioned outside the chamber. The neutron diagnostic arrangement was designed to study the features of laser-driven neutron beams for active interrogation. Two ^3^He thermal neutron coincidence well counters were placed outside the chamber for the detection of *β*-delayed neutrons, following neutron-induced fission, from active interrogation of nuclear material. These arise from neutron rich nuclei created following induced fission. One of the well counters contained a sample of nuclear material for active interrogation, while the other was kept empty to serve as a reference for background comparison. The counters used were high-level neutron coincidence counters (HLNCC-II)^24^ composed of a single ring of 18 ^3^He-filled proportional detectors embedded in high-density polyethylene. Neutrons impinging on the HLNCC-II detectors are spread out in time by thermalization and diffusion in the moderator assembly, enabling them to be counted in pulse mode. Both counters were located on the equator, closely straddling the central beam axis, defined by the laser propagation direction. It is important to emphasize that for the purpose of this paper, only data acquired in the reference (empty) detector are considered for the analysis, where no fissionable material that could lead to delayed neutron production is present. In addition, a single moderated ^3^He-filled proportional detector, was located ~90° off-axis. Furthermore, a neutron time-of-flight (nTOF) plastic scintillator detector was positioned along the central beam axis (the laser-propagation direction and the symmetry axis of the converter). This detector was used to measure the neutron energy distribution in the forward direction. The moderated ^3^He detector, as well as the HLNCC-II well counter, were covered in a thin (~1 mm thick) Cd foil to block the contribution of slow neutrons returning to the detectors after scattering in the room^25^. Prompt beam-neutrons produced by the laser that strike these detectors exhibit a characteristic 1/e die-away time of several tens of microseconds^25^. The die-away times of HLNCC-II and the single moderated ^3^He detector correspond to ~43 *μ*s and ~20 *μ*s, respectively. These time intervals are short compared to the delayed neutron production which typically extends over several hundreds of milliseconds or more. Thus, the delayed neutron signal can be clearly identified in these thermal-neutron detection systems.

Initial measurements were made to optimize empirically the neutron production and its directionality for active interrogation of nuclear materials. The characteristics of the neutron beam were investigated by changing target/converter configurations and varying the distance between them in a series of laser shots. The distance of the front face of the Be converter to the target was systematically increased to 3.6, 6.0, 8.0 and 12.0 mm over several shots and the results are reported here.

**Figure 2:**
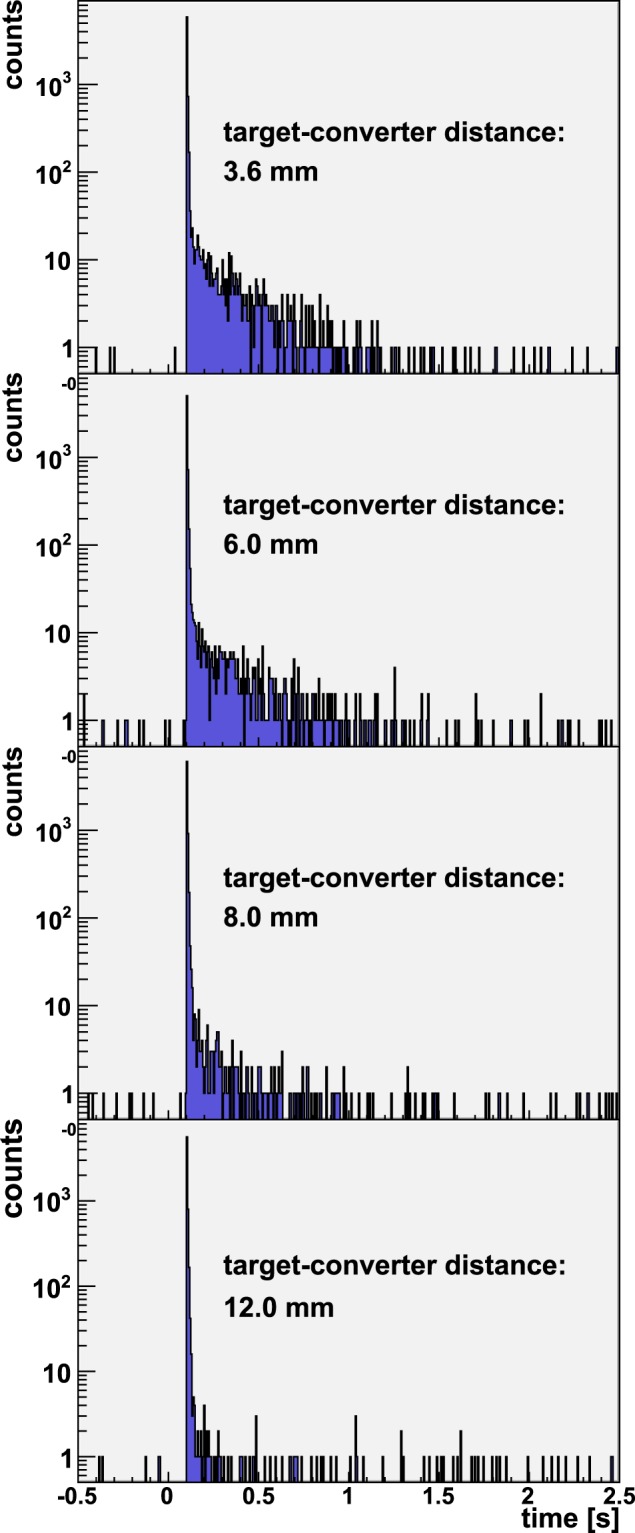
Delayed neutron production in the forward direction from ^9^Li decay is shown for four different shots in which Be ion-to-neutron converter was placed at different distances from the target as measured with the HLNCC-II detector. As the converter gets closer to the target, more high energy deuterons interact with the converter, hence increasing the ^9^Li production, and the subsequent delayed neutron decaying signal. These are the raw data before normalization for total neutron yield. Note that zero on the x-axis corresponds to the time of laser pulse and negative values represent background data acquired immediately before the shot.

**Figure 3:**
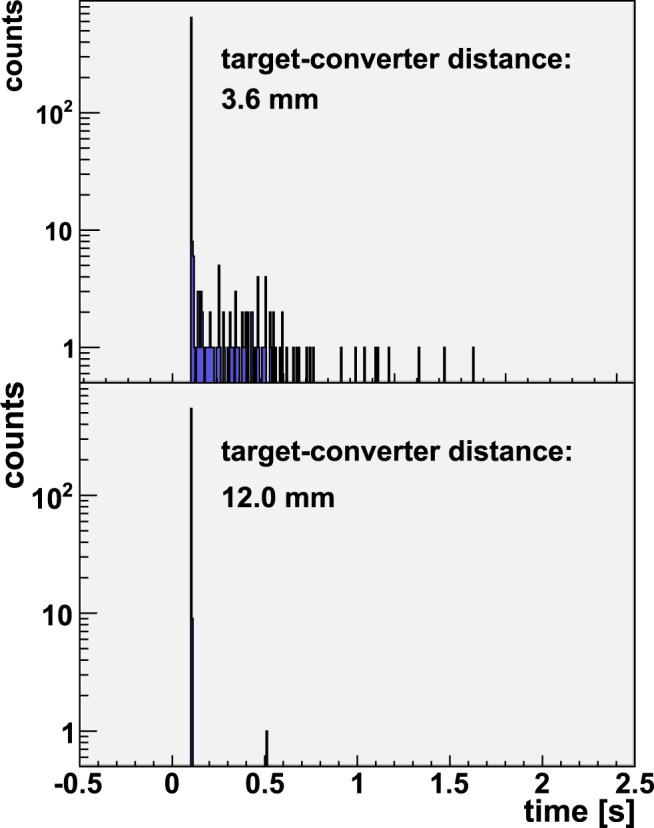
^9^Li delayed neutron spectra in the ^3^He neutron monitor located at ~90° from the forward direction shown for two shots with different converter-target distances. The ^9^Be converter catches high energy deuterons when at 3.6 mm, while at 12 mm, it misses most them. Delayed neutron emission from ^9^Li decay is expected to be isotropic, thus consistently the ^9^Li delayed neutron signal is measured by detectors located at ~90°.

**Figure 4:**
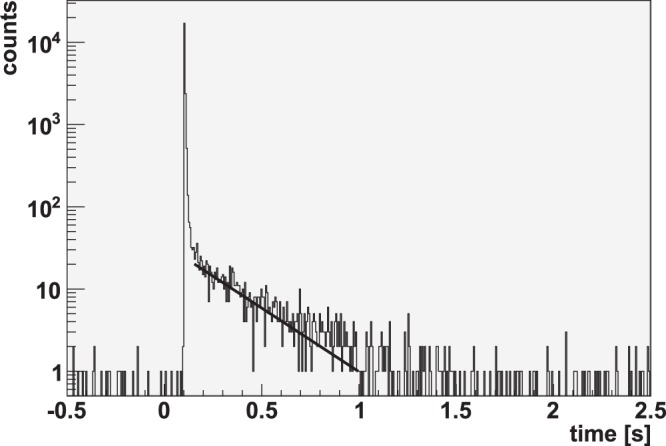
Summed time-interval distributions measured in the HLNCC-II counter for target/converter distances of 3.6, 6.0 and 8.0 mm. The decay constant (260 ± 20 ms) is calculated by a weighted least squares fit and the uncertainty (1 *σ*) by varying the fit region.

During the measurements in which the Be converter was placed at a separation of either 3.6, 6.0 or 8.0 mm, a delayed-neutron tail following the prompt neutron peak was observed in the ^3^He detectors not containing any nuclear material. So the origin of these neutrons could not be from induced fission. The tail was observed in the reference (i.e. empty) HLNCC-II well counter, located in the forward direction as well as in the single ^3^He detector located at ~90° off-axis, as shown in Figs 2 and 3, respectively. These figures show the time-interval distribution of the neutron detection recorded following the laser pulse. It can be seen that the tail decays completely in ~1 second. The 1/e-value (*τ*) life time extracted from these experiments corresponds to 260 ± 20 ms. This value was obtained by summing-up the time-interval distributions measured in the HLNCC-II well counter for target-to-converter distances of 3.6, 6.0 and 8.0 mm and performing an exponential fit to determine the slope of the decay (see Fig. 4). The extracted life time (*τ*) corresponds, within the measurement uncertainty, to the delayed neutron production via decay of ^9^Li with a published value of *τ* = 257.2 ms^20^. This delayed neutron production is expected to be isotropic, as confirmed by the comparison of the signals from the HLNCC-II and the ^3^He flux monitor, located at ~0° and ~90°, respectively.

Because of the die-away time characteristics of the ^3^He detectors used, the temporal signal produced directly by the laser-driven prompt neutron pulse dissipates approximately within a ms, since 1 ms corresponds to more than 20 times the 1/e-die-away period of the detector. In addition, return of the scattered thermal neutrons from the room is suppressed by the external Cd shield, and in any case it would not display the characteristic decay time of the ^9^Li decay. The contribution of these effects to the delayed neutron signature, observed over the period of 50–1000 ms, can therefore be excluded.

The ^9^Li produced in this experiment originates from high energy deuterons from the ^9^Be(d, 2p)^9^Li reaction, which has a threshold of 18.42 MeV^21^. There is a second possible reaction, ^9^Be(n, p)^9^Li, with a threshold of 14.26 MeV^22^, which could be caused by high energy neutrons coming from >30 MeV deuteron break-up reactions in the converter, so again the production is related to high energy deuteron production. (For completeness we can estimate the ratio of ^9^Li production from the two reactions. The ratio of (n, p)/(d, 2p) production is the product of the numbers of neutrons/deuterons, the cross-section ratio, and the interaction volume. The conversion efficiency, neutrons/deuterons, for deuterons above 15 MeV has been measured to be around 0.1%^4^ for this size Be converter. The ratio of cross-sections, (n, p)/(d, 2p) is not expected to exceed ~0.5^26^ and in the extreme case of parallel beams of particles the interaction volume for the neutrons could be the full length of the converter, whereas the deuteron interaction volume is limited to the deuteron range. This latter ratio is about 20. The overall ratio of (n, p)/(d, 2p) is therefore of the order of 1% and the (d, 2p) reaction is clearly the dominant one.)

**Figure 5:**
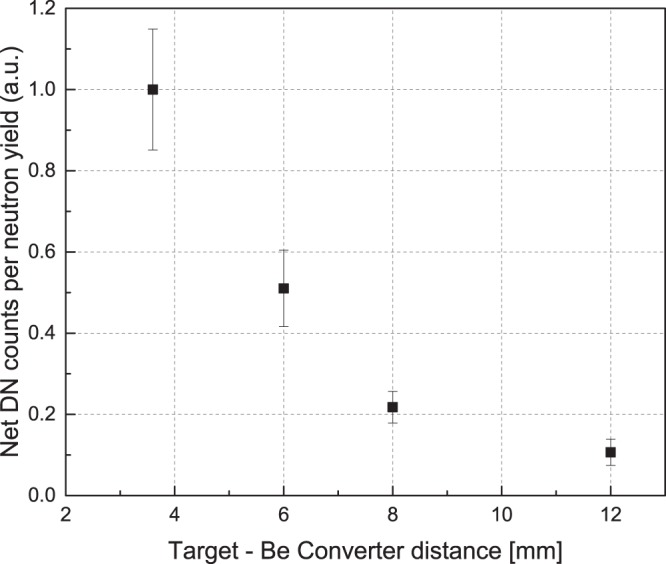
^9^Li delayed neutron (DN) production, normalized for the neutron yield (HLNCC-II at 0°), as a function of the distance between the Be back face and the target. The uncertainties are statistical only. The uncertainty on the 3.6 mm datum is larger than the others because it contains a correction factor for the orientation axis of the HLNCC-II detector.

We observed that as the distance of the converter to the CD_2_-target increases, the yield of the delayed neutrons, proportional to ^9^Li production, decreases. Figures 2 and 3 illustrate this trend. Therefore, at a large enough target-to-converter distance, the flux of the high-energy deuterons on the converter, that governs the ^9^Li production, approaches zero. The integral of the delayed neutron counts normalized to the laser neutron yield are shown in Fig. 5 for all of the target-converter distances. The delayed neutron counts displayed were integrated over an interval of 50 – 1000 ms after the laser pulse. If the observed delayed neutron production from ^9^Li decay were caused by a mechanism involving a quasi-parallel beam of forward directed deuterons above the ^9^Li production threshold striking the Be converter, the delayed neutron yield would be comparatively insensitive to the distance between the CD_2_ laser target and the Be converter. This is inconsistent with our observations. (It can be anticipated that at a sufficiently close distance between the converter and the deuteron source most of the deuterons would hit the Be and the dependence versus separation would level off. Such distances (less than 3.6 mm) were not investigated in this experiment in order to avoid Be damage and contamination inside the chamber.) These observations and their scaling imply a large divergence angle for the high-energy deuteron production responsible for the ^9^Li (see Discussion). Although these data enable us to estimate a lower bound for the cone angle of the fast deuterons, they don’t provide information about the energy distribution within that cone, except that it exceeds the ~20 MeV threshold.

**Figure 6:**
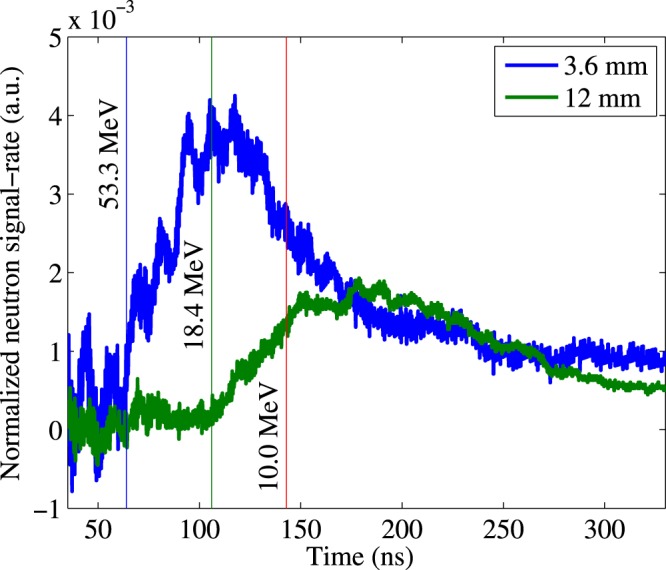
Neutron time-of-flight spectra for two shots with the Be converter placed at 3.6 and 12.0 mm from the target, respectively. The vertical lines mark the highest observed energy of the neutron distribution of ~55 MeV and ~18 MeV for 3.6 mm and 12 mm distances, respectively. A further vertical line marks 10 MeV neutron energy. Higher energy neutrons (to the left of this line) can only be produced by breakup of high energy (>20 MeV) deuterons.

**Figure 7:**
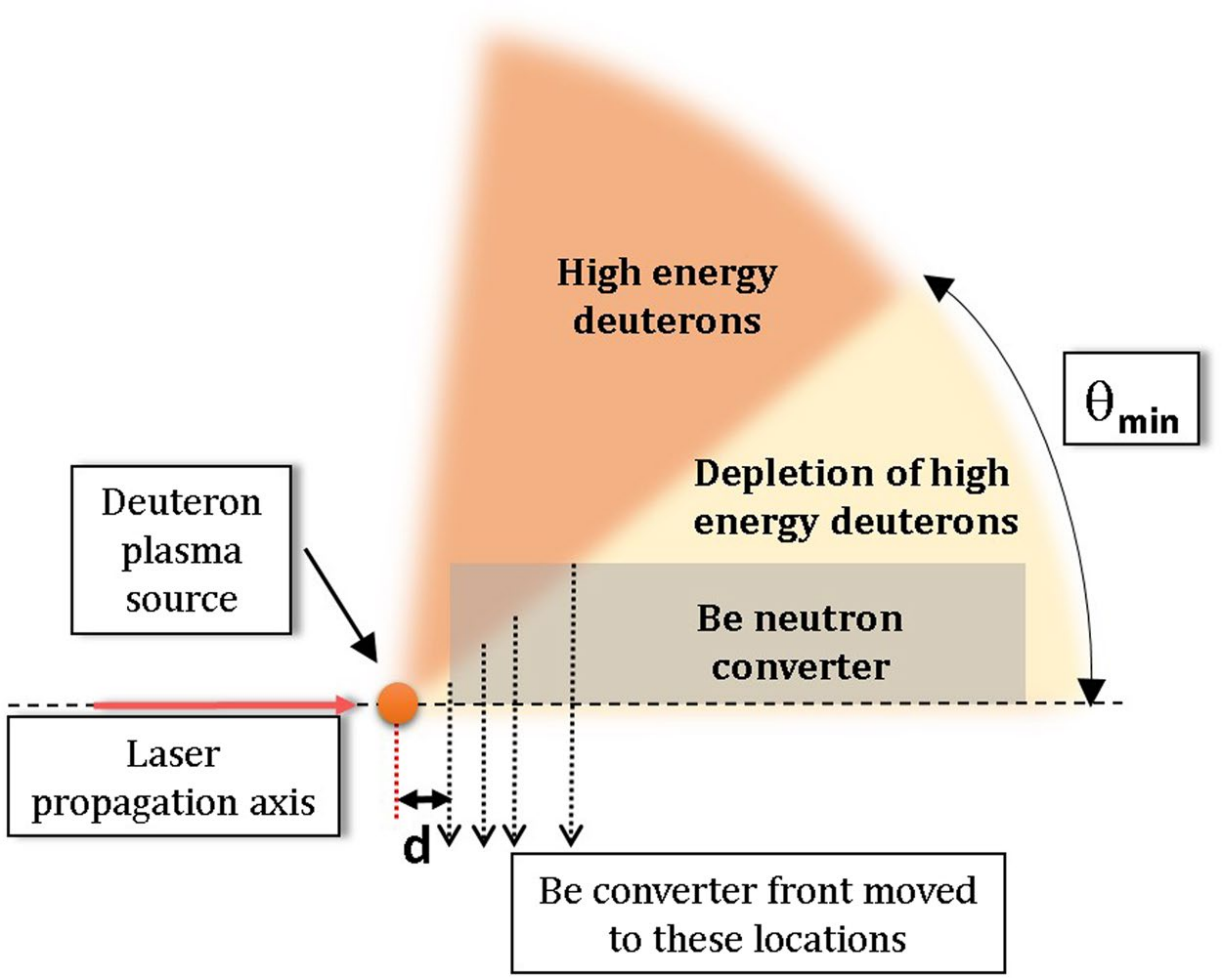
Illustration of the morphology of the laser-driven deuteron beam deduced from the neutron measurements in this manuscript. Deuterons above the ^9^Be(d, 2p)^9^Li reaction threshold come out essentially from a point (the laser-plasma) irradiating the Be cylinder used as the neutron converter. The converter is separated from the laser plasma at a distance d, which is varied in the experiment. The deuterons (with energies above the threshold) are severely depleted from the inner cone defined by the angle *θ*_*min*_. Therefore the ^9^Be(d, 2p)^9^Li can only happen in the converter volume with a radius outside the *θ*_*min*_ cone and a depth within the deuteron range in Be. The neutron yield from these high energy deuterons is proportional to that volume.

Independent data from the nTOF detector located on the central beam axis provides insight on the energy distribution of the fast deuterons within the cone discussed above. nTOF data in Fig. 6 show the dependence of the neutron beam energy on the target-to-converter distance. Specifically, it shows time-of-flight spectra of the prompt neutron beam arising from the deuterium disintegration in the converter for target-to-converter distances of 3.6 and 12 mm. The deuterons of interest for ^9^Li production (>20 MeV), when undergoing break-up, produce neutrons with energies of ~10 MeV or more. Therefore in Fig. 6, we compare the neutron spectra below 143 ns that corresponds to neutrons with energies greater than 10 MeV. If the deuteron spectra were the same at all angles we would expect the prompt neutron spectra in nTOF to be self-similar for any separation. Instead, Fig. 6 shows a spectrum severely depleted, by an order of magnitude, in neutrons above 10 MeV, relative to those below 10 MeV when the converter separation is 12 mm, compared to the spectrum at the minimum separation of 3.6 mm. This lack of high energy neutrons implies a lack of fast deuterons impinging on the Be converter which suggests an angular distribution of the fast deuterons that is depleted along the central axis. This is contrary to expectations from most laser-driven ion accelerations mechanisms. The morphology of the deuteron beam inferred from our measurements is illustrated in Fig. 7. Deuterons above the ^9^Be(d, 2p)^9^Li threshold energy propagate from the nearly point-like laser plasma but are severely depleted within the inner cone defined by the half-angle *θ*_*min*_. (Note that this experiment does not give any estimation of any potential maximum angle of deuteron distribution). In the experiment the target converter surfaces were normal to the laser propagation axis^4^. *θ*_*min*_ has to be large enough for the inner depletion cone to include nearly the full diameter of the converter at d = 12 mm in order to explain the low yield at that separation. However *θ*_*min*_ cannot be too large, otherwise the ^9^Li neutron yield in Fig. 5 would fall even faster than observed for the largest target-converter separations (8 and 12 mm). As a rough estimate we assume that all fast deuterons barely miss the converter at the 12 mm distance to get *θ*_*min*_ = arctan(10/12) = 40°. A better yet simple model of the yield as a function of target-converter distance can be made based on the volume of the converter that is accessible to the high energy deuterons. This converter volume is defined in radius as lying outside the *θ*_*min*_ cone, and in depth by the range of the deuterons in solid Be. We note that a representative energetic deuteron (~50 MeV)^3,4^ would penetrate ~0.85 cm into the Be converter. For our simple model, we assume the high-energy deuteron angular flux is zero within the *θ*_*min*_ cone, and uniform at greater angles. It has then two free parameters: *θ*_*min*_ itself and a scaling factor. That model describes the data of Fig. 5 with a value for *θ*_*min*_ of 44°. This is contrary to expectations from most laser-driven ion acceleration mechanisms.

The ring-like morphology for the fast deuteron population is consistent in light of prior simulations and experiments on laser-driven C^6+^ beams in the BreakOut Afterburner (BOA) regime, in the relativistic transparency regime where Trident experiments are known to operate. We note that deuterons have the same charge to mass ratio of fully ionized carbon. 3D simulations of the interaction of an intense laser with diamond nanofoils resulting in BOA acceleration discussed in ref.^23^, which are defined by the charge to mass ratio, show such a ring-like structure for the high-energy portion of the C^6+^ spectrum. The experiments in ref.^23^ carried out on the Trident laser with diamond nanofoils also showed an increasingly ring-like emission versus angle for increased C^6+^ ion energy, albeit at a smaller angle ~10°. Although, deuterated plastic and diamond targets are different in their chemical structure and density, with proper optimization of the target thickness to yield a similar time during the laser pulse when the laser target becomes relativistically transparent, it is not unreasonable to expect similar behavior. However, the response of the two materials to the laser pre-pulse and the laser hydrodynamic disassembly would not be identical, so quantitative differences are to be expected.

## Discussion

An intense and energetic deuteron beam is generated by irradiating a sub-micron thick polyethylene target with the intense Trident short-pulse laser. The deuterons produce neutrons by impinging on a beryllium cylinder placed in the beam path. This technique produces a higher forward-directed neutron flux per unit laser energy than any alternative available so far. Our neutron detectors, which were designed for active interrogation of special nuclear material, detected delayed neutrons from ^9^Li decay with its characteristic mean life time of 257.2 ms. We attribute the ^9^Li production primarily to ^9^Be(d, 2p) reactions driven by energetic deuterons above the reaction threshold of 18.42 MeV.

The delayed neutron yield gives a direct on-line measure of the number of high energy deuterons impinging on the beryllium converter. The measured delayed neutron yield from the ^9^Li decay is observed to decrease steeply as the separation between the laser target and neutron converter is increased. That dependence is consistent with the high energy portion of the deuteron beam being emitted from the laser target at angles (relative to the central axis) greater than ~40°. Moreover, data from the neutron time of flight diagnostic along the central axis have been used to measure the energy spectrum of the prompt beam neutrons created by deuterium disintegration in the converter, for various laser-target to converter separations. Those neutron spectra have been used to estimate the relative abundance of the fast deuterons, i.e., above the ^9^Li production threshold. It is found that the flux of those fast deuterons is severely depleted along the axis. These observations are consistent with the hypothesis that the fast deuteron population is being emitted in a ring-like distribution around the central axis. This hypothesis is qualitatively consistent with one of the documented unique signatures of the BOA mechanism. Thus, our work provides independent experimental confirmation of a key signature of the Breakout Afterburner ion acceleration mechanism: the divergence of the highest energy portion of the ion beam from relativistic collective laser-plasma effects. Our methodology is completely independent and distinct from the one typically used in laser-plasmas^23,27^. Our results add important information to the ongoing debate about the dominant processes in the interaction of intense lasers with thin foils. However, in these particular laser targets this BOA signature turned out to be much larger than expected based on past targets and prior limited observations on these targets with ion spectrometers^4^. This demonstrates both the striking effectiveness of this complementary technique as well as the capability of quantitative reevaluation when laser targets are changed, even when the same basic plasma physics remains operative.

In this paper, we explain the use of a specific nuclear reaction as a diagnostic tool to measure important distinguishing features of competing laser-plasma ion acceleration mechanisms. This method provides a novel and direct diagnostic capability. Our results demonstrate the previously unrecognized power of selected nuclear reactions as a highly specific diagnostic for deuteron beam spectra and morphology. In the case of a Be converter, *β*-delayed neutron measurements, from ^9^Li decay, offer an attractive alternative relative to other diagnostics tools because they can be used continuously throughout any experimental campaign. Note that, in these experiments, the converter obstructs and thus complicates the use of other possible beam diagnostics. The nuclear reaction based method we offer uses the converter itself for diagnostic purposes, becoming a seamless part of the experiment and opening new possibilities in the field. As demonstrated in Fig. 3, a simple moderated ^3^He-filled proportional counter may be used to detect the delayed neutron signature and provide a flux monitoring capability. Nuclear diagnostic methods such as this one, could also be very useful to resolve ambiguous results with particle spectrometers when ions with similar charge to mass ratios are involved.

The measured integral delayed-neutron counts, corrected for the efficiency of the ^3^He detector and the ^9^Li production cross section, could be used to determine the fraction of high energy deuterons in every shot that strike the converter. (These higher energy deuterons produce higher energy prompt neutrons.) In general, the quantitative power of the technique would be greatly increased if the experimental reaction cross section was known as a function of energy^21,26^ from threshold to around 100 MeV. Therefore, our work provides an impetus for further work to measure the ^9^Be(d, 2p) cross section. In the development of fieldable laser-driven active interrogation of nuclear material, the fact that we have detected *β*-delayed neutrons from a non-fission source has important implications: (1). They can be exploited to monitor the fraction of fast neutron interrogation of nuclear materials (this could be used to characterize the nuclear material due to the fission cross section dependence with neutron energy), (2). They are a potential source of neutron background. The latter needs to be taken into account in order to avoid biases in the estimates of nuclear material amounts^6^. In general all the findings reported here will be exploited to guide future experimental studies and to design neutron based applications of the laser-beam source.

## Methods

### Laser system

The experiments were carried out at the LANL 200 TW Trident laser facility. The Trident laser facility provides a very high-contrast (10^−8^ at −10 ps)^9^ laser pulse of energy ~80 J. The target chamber radius is 1 m and its wall is 20 mm thick stainless steel, with 25 mm thick Al flanges located around the chamber.

### Neutron diagnostics

The neutron counters used were High-Level Neutron Coincidence counters (HLNCC-II)^24^ composed of a single ring of 18 ^3^He-filled proportional detectors (4 atm ^3^He partial pressure;active length 50.8 cm, diameter 2.5 cm) embedded in high-density polyethylene. The count rates from both detectors were acquired in list mode (with a time stamp), with 10 ns time resolution and analyzed in the form of time-interval distributions of the neutron detection times, following the laser trigger pulse. Several bubble detectors(BTI) were distributed around the chamber to measure laser generated neutron yield in multiple directions^3,4,6^.

### Neutron time-of-flight

A neutron time-of-flight (nTOF) plastic scintillator detector (10 cm diameter, 1.88 cm thick NE02 plastic scintillator coupled to 12.5 cm Hamamatsu R1250A PMT) was positioned at 6.2 m away from the converter along the central beam axis (the laser-propagation direction and the symmetry axis of the converter). This detector was used to measure the neutron energy distribution in the forward direction. It was located outside the building to detect the high-energy portion of the neutron spectrum with better resolution^3^.
